# Decitabine modulates dopamine agonist sensitivity in gh3 rat pituitary tumor cells via Trim7

**DOI:** 10.3389/fendo.2026.1857666

**Published:** 2026-09-17

**Authors:** Xu Zhang, Tengwu Chang, Jinyong Li, Hua Gao, Wenju Bai, Hao Yue, Cheng Zhang, Peikun Zhang, Yuanyan Ge, Jichao Wang

**Affiliations:** 1 Xinjiang Second Medical College, Karamay, China; 2 The Fifth Clinical Medical College, Henan University of Chinese Medicine (Zhengzhou People’s Hospital), Zhengzhou, China; 3 Department of Cell and Biology, Beijing Neurosurgical Institute, Capital Medical University, Beijing, China; 4 Neurosurgical Department, People’s Hospital of Xinjiang Uygur Autonomous Region, Urumqi, China

**Keywords:** decitabine, differential expression, drug-resistant, pituitary prolactinoma, TRIM7

## Abstract

**Introduction‌:**

Pituitary prolactinoma is the most prevalent subtype of functional pituitary adenoma, for which dopamine agonists (DAs) have been established as the universal first-line therapeutic regimen in clinical practice. Approximately 10–25% of patients exhibit poor therapeutic responsiveness to DAs, presenting with persistent hyperprolactinemia and uncontrolled tumor growth, while the molecular mechanisms underlying this drug resistance phenotype remain incompletely characterized.

**Methods:**

In the present study, GH3 rat pituitary tumor cells were employed as the in vitro experimental model to investigate whether the DNA methyltransferase inhibitor decitabine could modulate cellular sensitivity to dopamine agonists, and to further elucidate the associated potential molecular basis. Transcriptome sequencing coupled with systematic bioinformatics analysis was conducted on GH3 cells after decitabine intervention to screen candidate differentially expressed genes, from which Trim7 was selected as the key target for subsequent functional validation.

**Results:**

Functional assays demonstrated that Trim7 overexpression significantly enhanced cabergoline-induced apoptosis in GH3 cells. A concomitant reduction in cell proliferation was also observed in the Trim7 overexpression group compared with the empty vector control, although this difference did not reach statistical significance.

**Discussion‌:**

Collectively, these data establish a significant correlative relationship between Trim7 upregulation and improved dopamine agonist sensitivity in GH3 cells. Complementary loss-of-function experiments are still required to define the definitive causal role of Trim7 in this regulatory axis. The present findings provide preliminary evidence for the involvement of epigenetic regulation in dopamine agonist resistance, and suggest that Trim7 may serve as a promising potential therapeutic target for future intervention strategies in drug-resistant prolactinomas.

## 1 Introduction

The annual incidence of pituitary adenoma is as high as 7.5–15/100,000 people (1, 2), and pituitary prolactinoma is the most common pituitary adenoma with endocrine function, accounting for approximately 45–70% of all pituitary adenomas (3). With the improvement of people’s health awareness and the improvement of diagnostic technology, imaging equipment, and surgical level, the actual number of patients diagnosed clinically is significantly higher than the above data. The clinical manifestations of prolactinoma are female infertility, amenorrhea, and galactorrhea caused by high PRL, decreased libido and sexual function, infertility in men, and oppressive symptoms, including headache, vision loss, and visual field damage. These symptoms seriously affect the quality of life of patients. In recent years, the number of related deaths and disabilities has also been increasing, which has brought a heavy burden to society and families (1).

At present, for pituitary prolactinomas, drug therapy is the main treatment, and dopamine receptor agonists (DAs) are the preferred treatment method. Bromocriptine and cabergoline are the DAs used clinically as first-line drugs. DA drugs can specifically bind to dopamine receptors on the surface of prolactinoma cells, thereby inhibiting the production and secretion of PRL, and they can mediate the shrinkage of tumor volume. However, in current clinical treatment, approximately 10–25% of patients are not sensitive to DA drug treatment; that is, they have drug-resistant prolactinomas (DARPs). At present, there are no strict regulations on the definition of DARPs across the world. From the perspective of endocrine, the main point is “failure to normalize serum PRL level” (2). The degree of tumor volume reduction is generally defined by administering the maximum conventional dose of drugs (15 mg/day of bromocriptine or 3 mg/week of cabergoline) continuously for at least 3 months, with the expectation of achieving a reduction in tumor size of at least 50%. At present, in approximately 17% of patients, the PRL level cannot be reduced to the normal range after cabergoline treatment.

Currently, non-drug treatments for patients with DARPs mainly include surgery and radiotherapy. However, the effect of surgical treatment in patients with DARPs is poor. Hamilton et al. reported that only 36% of 61 cases of DARPs achieved normal postoperative PRL levels following surgery (3). Epidemiological studies showed that DARPs are more likely to invade the cavernous sinus (i.e., approximately 71% of DARPs), while only 10% of sensitive prolactinomas invade the cavernous sinus (4). Radiation therapy, such as Gamma Knife, can be used for residual DARPs after surgery. Unfortunately, prolactinoma is the least sensitive functional pituitary adenoma to radiotherapy. The 4-year PRL hormone remission rate is 18%, while the remission rate after Gamma Knife treatment is only 16% (5). Therefore, the effect of surgery and radiotherapy on DARPs remains poor, proving that clinical treatment is very difficult.

Therefore, as a supplement to the existing DARP treatment scheme, it is necessary to develop new therapeutic drugs according to the mechanism of DARPs. Decitabine (DAC) is a DNA methyltransferase (DNMT) inhibitor, which can induce DNA demethylation, such that the key genes that play a vital role in cell differentiation and proliferation can be restored, thereby playing an antitumor role (6). The previous research results of our team showed that, in rat prolactinoma specimens, the hypermethylation of the WIF-1 promoter −111 bp site led to a decrease in the expression of WIF-1 in tumor cells and the use of DAC to treat MMQ. After treatment, the expression of WIF-1 increased by 6.56 ± 1.45 times (p < 0.001), and the cell proliferation was also significantly inhibited, reaching 37.8% ± 4.3% (p < 0.001) (7). In addition, we found that, after treating GH3 cells with different concentrations of DAC, the cell proliferation decreased in a concentration- and time-dependent manner. GH3 cells were treated with 40 nM of DAC for 72 h, and the inhibition rate of cell proliferation was the largest, reaching 87.6% ± 2.2%.

In this study, we treated GH3 rat pituitary tumor cells with decitabine and performed high-throughput transcriptome sequencing and bioinformatics analyses to identify differentially expressed genes, among which Trim7 was selected for further investigation. Trim7 was subsequently overexpressed in GH3 cells, followed by cabergoline treatment to investigate whether elevated Trim7 expression was associated with altered cellular responsiveness to cabergoline.

## 2 Materials and methods

### 2.1 GH3 rat pituitary tumor cell line

The rat GH3 cell line was used in the experiment, purchased from Procell Life Science and Technology Co., China, Wuhan. To resuscitate and extract the RNAs of GH3 cells, we performed QPCR detection and agarose gel identification, confirming that this batch of GH3 cells was of rat origin.

### 2.2 Cell culture and medication intervention

The sample GH3 cells were divided into six groups, with three DAC intervention groups (10 μmol/L) and three blank control groups for follow-up experiments.

The GH3 cells were cultured in phenol red-free DMEM culture supplemented with 10% (v/v) charcoal-stripped fetal bovine serum in a humidified incubator at 37 °C in 5% (v/v) CO2. The culture medium was replaced every day.

Log-phase GH3 cells (>99% viable) were plated into six-well culture plates (~1 × 10^6^ cells/well) and were treated with decitabine solution (10μM) or with the corresponding solvent (DMSO). After treatment for 72 h, the cells were harvested for RNA transcription sequencing analysis.

### 2.3 RNA extraction and sequencing

Total RNAs were extracted from the GH3 cells using TRIzol Reagent (Invitrogen) following the method of Chomczynski et al. (https://doi.org/10.1006/abio.1987.9999). DNA digestion was carried out after RNA extraction by DNaseI. RNA quality was determined by examining A260/A280 with a NanodropTM OneC spectrophotometer (Thermo Fisher Scientific Inc, Waltham Massachusetts, USA)). RNA integrity was confirmed by 1.5% agarose gel electrophoresis. Qualified RNAs were finally quantified by Qubit3.0 using a QubitTM RNA Broad Range Assay Kit (Life Technologies, Q10210, Waltham, MA, USA).

miRNAs: First, 2 μg of total RNAs were used for small RNA cDNA library preparation with a Balancer NGS Library Preparation Kit for small/microRNAs (GnomeGen, San Diego, CA, USA) following the manufacturer’s instructions. Briefly, the RNAs were ligated to 3’ and 5’ adaptors and sequentially reverse-transcribed to cDNA, before being PCR-amplified. The whole library was applied to 12% native PAGE gel electrophoresis, and bands corresponding to microRNA insertion were cut and eluted. After ethanol precipitation and washing, the purified small RNA libraries were quantified and stored at −80 °C before sequencing.

mRNAs/lncRNAs: First, 2 μg total RNAs were used for stranded RNA sequencing library preparation using a KCTM Stranded mRNA Library Prep Kit for Illumina (Catalog NO. DRhealth Co., Ltd.,Wuhan, China) following the manufacturer’s instruction. PCR products corresponding to 200–500 bp were enriched and quantified.

For high-throughput sequencing, the libraries were prepared following the manufacturer’s instructions and applied to th08402, Wuhan Seqe Illumina NovaSeq 6000 system(Illumina, San Diego, CA, USA) for 150 nt paired-end sequencing.

### 2.4 Differential expression analysis

The R Bioconductor package edgeR was utilized to screen out the differentially expressed genes (DEGs). A p-value for correction of <0.05 and a fold change of >2 or <0.5 were set as the cutoff criteria for identifying DEGs.

### 2.5 Expression network of lncRNA–miRNA–mRNA differential genes

The lncRNA, miRNA, and mRNA expression levels in cells before and after DAC intervention were quantified. After DElncRNAs, DEmiRNAs, and DEmRNAs were obtained; magia (miRNA and gene integration analysis tool) was used for joint analysis of the gene interactions. The differentially expressed genes were uploaded separately to map the interaction network. The differentially expressed genes shared by the lncRNA–miRNA–mRNA network and correlated with one another were obtained, and DEG verification analysis was performed.

### 2.6 Gene overexpression and drug intervention

The cells were cultured in basic medium with 10% fetal bovine serum and 1% penicillin and streptomycin (100 U/mL) at 37 °C and 5% CO2 saturation humidity. All of the cells used in the experiment were in the logarithmic growth stage. After the normal cultured cells were taken, the original culture medium was sucked away; then, the cells were washed with PBS and digested with pancreatic enzyme for 1–3 min. Next, the digestion was terminated, and single cells isolated with a pipette gun, before 15 μL of cell suspension was taken for counting. The remaining cell suspension was centrifuged at 1000 RPM for 5 min and then resuspended with medium, before adding 1 × 10^5^ cells to each well of a 24-well plate. Three replicates per group were cultured overnight at 37 °C with 5% CO2. After overnight culture, plasmid transfection, quality inspection, and drug intervention were performed as follows:

1. The plasmid volume of the above transfection quantity was taken, and the serum-free medium of the above dilution volume was added, before incubating for 5 min.
2. The transfection volume of Lipofectamine 2000 Transfection Reagent (11668019, Invitrogen, Carlsbad, CA, USA) was added to the serum-free medium at the above dilution volume and incubated for 5 min.
3. The solutions from step (1) and step (2) were combined and incubated for 20 min.
4. The above mixture was uniformly added to the cell culture medium and cultured in a 5% CO2 and 37 °C incubator for 6 h; then, the corresponding fresh and complete media were replaced and cultured in a 5% CO2 and 37 °C incubator for 24 h. Following this, the medium containing 10 μM cabergoline was treated for 24 h.
5. Transfected cells were harvested after 48 h for RT-qPCR and Western blotting analysis.

### 2.7 Quality detection of transcription

Western blotting: The GH3 cells were lysed in ice-cold RIPA Buffer (PR20001, Proteintech, Wuhan, China) supplemented with a protease inhibitor cocktail (4693116001, Sigma, MO, USA) and incubated on ice for 30 min. The samples were boiled for 10 min in boiling water with protein loading buffer (P1040, Solarbio, Beijing, China), loaded onto 10% SDS-PAGE gel, and transferred onto 0.45 mm PVDF membranes (ISEQ00010, Millipore, Billerica, MA, USA). The PVDF membranes were then blocked for 1 h at room temperature and incubated overnight at 4 °C with primary antibodies against FLAG tag (anti-FLAG, 1:1000, antibody produced in mice, 66008-3-lg, Proteintech, Wuhan, China) and GAPDH (1:10,000, antibody produced in mice, 60004-1-lg, Proteintech, Wuhan, China), followed by incubation with horseradish peroxidase-conjugated secondary antibodies (anti-rabbit, 1:10,000, SA00001-2, Proteintech, China or anti-mouse, 1:10,000, AS003, ABclonal, Wuhan, China) for 45 min at room temperature. Then, the membranes were visualized using enhanced ECL reagent (P0018FM, Beyotime, Shanghai, China) through chemiluminescence.

RNA quality analysis: Total RNA extraction was achieved using TRIZOL (15596-018, Ambion, Waltham, MA, USA). The RNAs were further purified with two phenol–chloroform treatments and then treated with RQ1 DNase (M6101, Promega, Madison, WI, USA) to remove DNA. The quality and quantity of the purified RNAs were determined by measuring the absorbance at 260/280 nm (A260/A280) using an Ultrafine spectrophotometer (N50 touch, IMPLEN, München, Germany). The integrity of the RNAs was further verified by 1.0% agarose gel electrophoresis.

Assessment of gene expression with qRT-PCR: cDNA synthesis was carried out using a reverse transcription kit (R323-01, Vazyme, Nanjing, China) at 42 °C for 5 min, 37 °C for 15 min, and 85 °C for 5 s performed on the thermocycler (T100, Bio-Rad, Hercules, CA, USA). qPCR was performed on ABI QuantStudio 5(Thermo Fisher Scientific Inc, Waltham Massachusetts, USA)), followed by denaturing at 95 °C for 10 min, 40 cycles of denaturing at 95 °C for 15 s, and annealing and extension at 60 °C for 1 min. Each sample had three technical replicates. The concentration of each transcript was then normalized to that of GAPDH (glyceraldehyde-3-phosphate dehydrogenase), and the mRNA level was determined using the 2−ΔΔCt method for analysis. Statistical analyses were performed using GraphPad Prism 8.0. Two-group comparisons for qRT-PCR, cell proliferation and cell apoptosis assays were conducted using two-tailed unpaired Student’s t-test. All functional experiments were performed with n = 3 independent biological replicates. All data are presented as mean ± SEM. The primers for the quantitative (q)PCR analysis are presented in **Supplementary Table 1**.

### 2.8 Cell proliferation and apoptosis assays

The cell proliferation assay was conducted using Cell Counting Kit-8 (CCK-8, 40203ES76, Yeasen, Shanghai, China). Briefly, the GH3 cells were seeded at 100,000 cells/well in a 24-well culture plate. The cells in the control and experimental groups were treated accordingly, and vials without cells were used as blank controls. Following culture for 48 at 37 °C and 5% CO2, 10 μL of CCK-8 solution was added to the culture medium and incubated for an additional 3 h at 37 °C. Following this, the optical density of the cells was measured using a microplate reader (ELX800, Biotek, Santa Clara, CA, USA) at an absorbance of 450 nm. The cell proliferation rate was calculated using the following formula: proliferation rate = (experimental OD value − blank OD value)/(control OD value − blank OD value) × 100%.

Flow cytometric analysis of cell apoptosis: To detect cell apoptosis, an Annexin V–APC/7-ADD apoptosis detection kit (40304ES60, Yeasen, Shanghai, China) was used according to the manufacturer’s instructions. Specifically, the GH3 cells were seeded into 12-well plates, cultured for 24 h, transfected with plasmid for 24 h, and treated with cabergoline for 24 h. The treated and control cells were mixed with 5 μL of Annexin V–APC and incubated at room temperature in the dark for 5 min, with 10 μL of PI reagent incubated for 5 min as described above. Then, the samples were subjected to flow cytometry (FACSCanto, BD, Franklin Lakes, NJ, USA) analysis to detect the cell apoptosis levels.

## 3 Results

### 3.1 Transcriptome profiling of the mRNA expression changes after DAC treatment

After obtaining the corresponding RNA sequencing results, we conducted gene differential expression analysis. First, sample correlation analysis was carried out, during which we analyzed the correlation between two samples as a function of the gene expression in each sample; that is, we checked the correlation among the gene expression levels in the different samples. Then, we conducted sample correlation PCA analysis as a function of the FPKM value of all detected mRNAs (**Figure 1A**). Second, edgeR software was used to compare the samples. The results indicated that 625 mRNAs were significantly differentially expressed between the DAC treatment and control groups, including 532 upregulated and 93 downregulated mRNAs (**Figure 1B**). All significantly differentially expressed genes were pooled, and a heatmap was generated according to their expression levels (**Figure 1C**).

**Figure 1:**
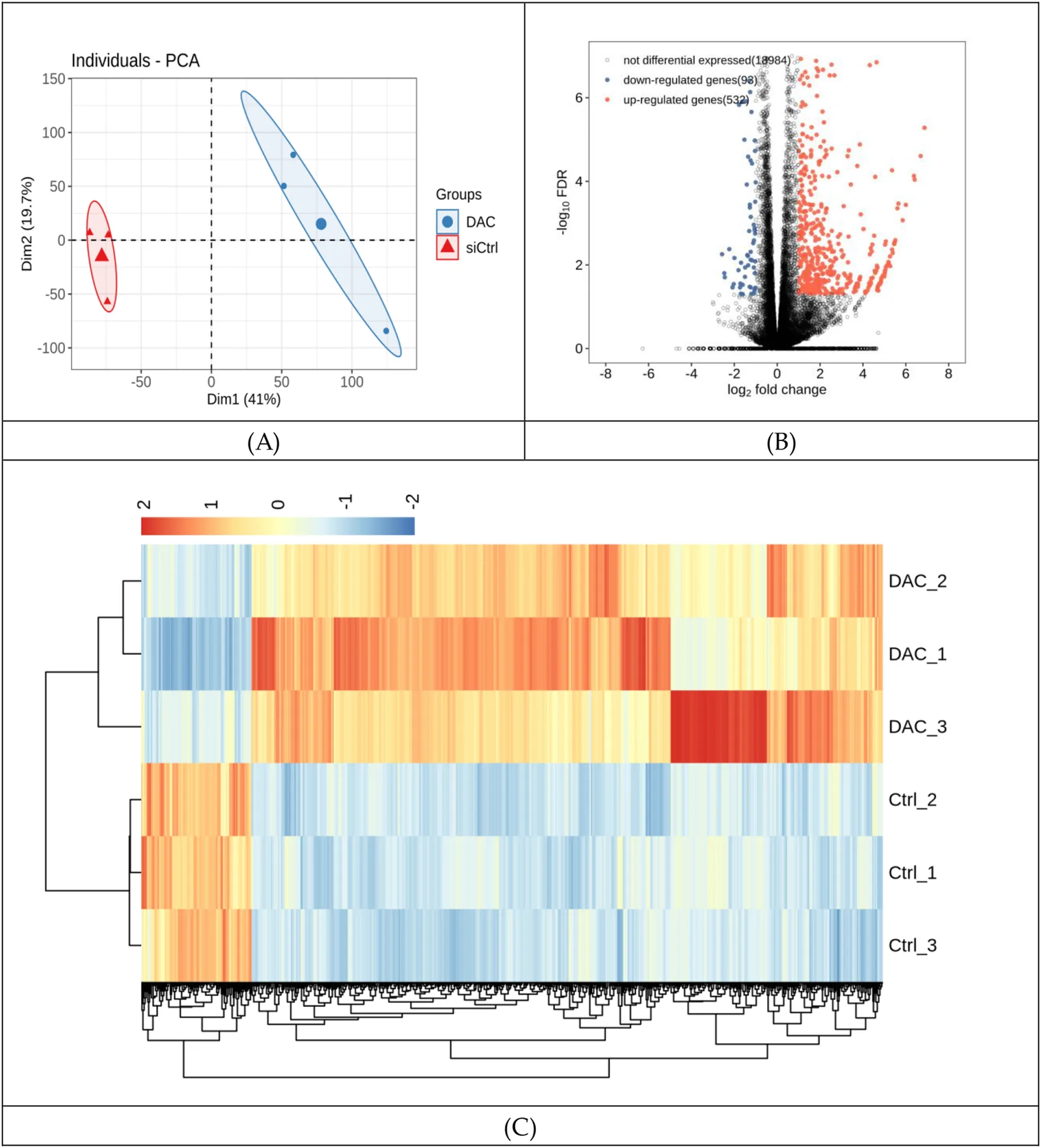
Transcriptome profiling of the mRNA expression changes in the GH3 cells after DAC treatment. **(A)** PCA based on the FPKM values of all of the detected genes. The ellipse for each group is the confidence ellipse. **(B)** Volcano plot showing all differentially expressed genes (DEGs) analyzed by edgeR (FDR < 0.05 and FC (fold change) ≥2 or ≤0.5). **(C)** Hierarchical clustering heatmap showing the expression levels of all DEGs. Data were obtained from n = 3 independent biological replicates per group.

### 3.2 lncRNA analysis of RNA-Seq in GH3 cells after DAC treatment

Similarly, after obtaining the corresponding RNA sequencing results, we conducted lncRNA differential expression analysis. First, sample correlation PCA analysis was performed according to the FPKM values of all detected lncRNAs (**Figure 2A**). Second, edgeR software was used to compare the samples, and the results showed that 163 lncRNAs were significantly differentially expressed between the DAC treatment and control groups, including 128 upregulated and 35 downregulated lncRNAs (**Figure 2B**).

**Figure 2:**
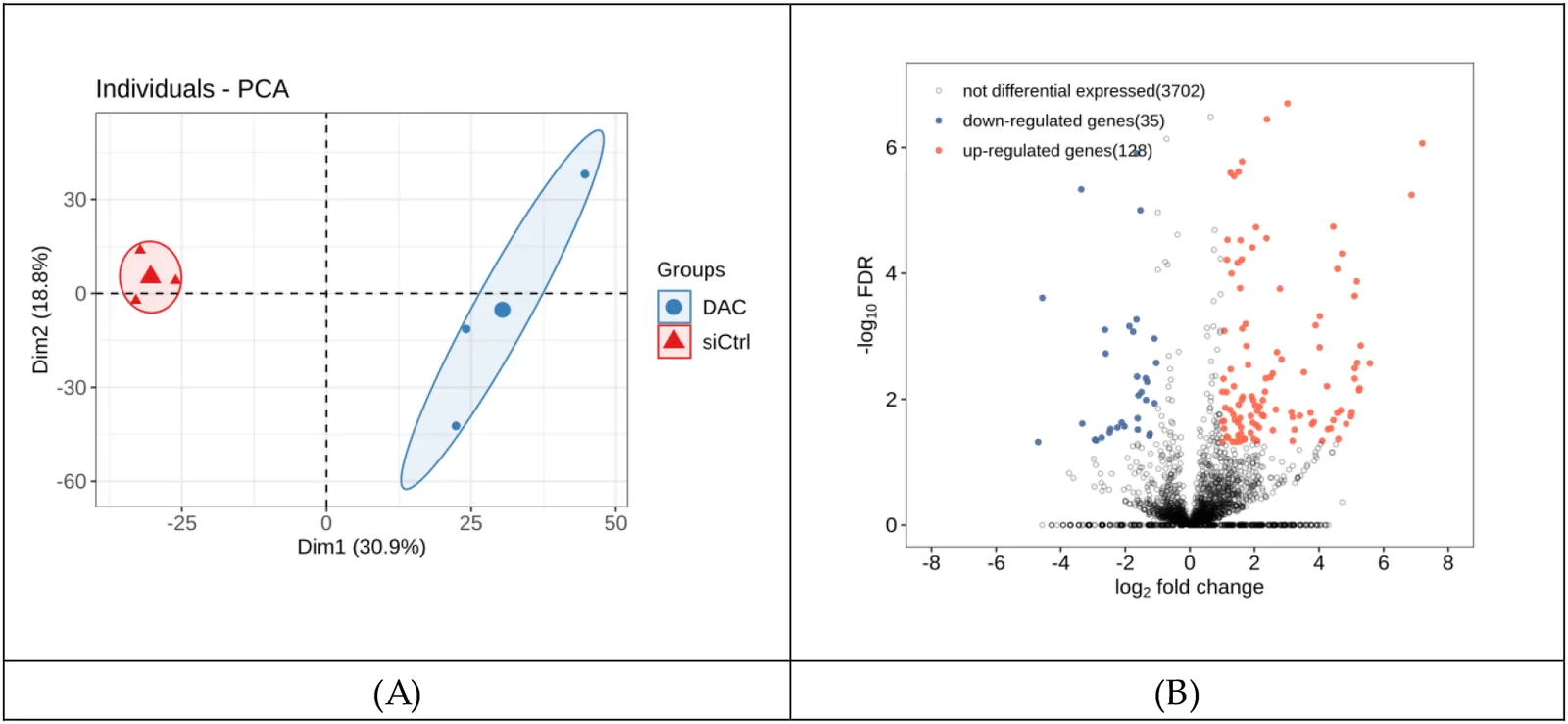
lncRNA analysis of RNA-seq in the GH3 cells after DAC treatment. **(A)** PCA based on the FPKM values of all detected lncRNA genes. The ellipse for each group is the confidence ellipse. **(B)** Volcano plot showing all differentially expressed lncRNA genes analyzed by edgeR (FDR < 0.05 and FC (fold change) ≥2 or ≤0.5). Data were obtained from n = 3 independent biological replicates per group.

### 3.3 Transcriptome profiling of the miRNA expression changes after DAC treatment

After obtaining the miRNA sequencing data, differential expression analysis and principal component analysis (PCA) were performed. PCA was conducted based on the FPKM values of all detected miRNAs (**Figure 3A**). Differential expression analysis was then performed using edgeR. A total of 116 miRNAs were identified as significantly differentially expressed between the DAC-treated and control groups, including 71 upregulated and 45 downregulated miRNAs (**Figure 3B**). The differentially expressed miRNAs were subsequently subjected to hierarchical clustering analysis, and a heatmap was generated based on their expression profiles (**Figure 3C**).

**Figure 3:**
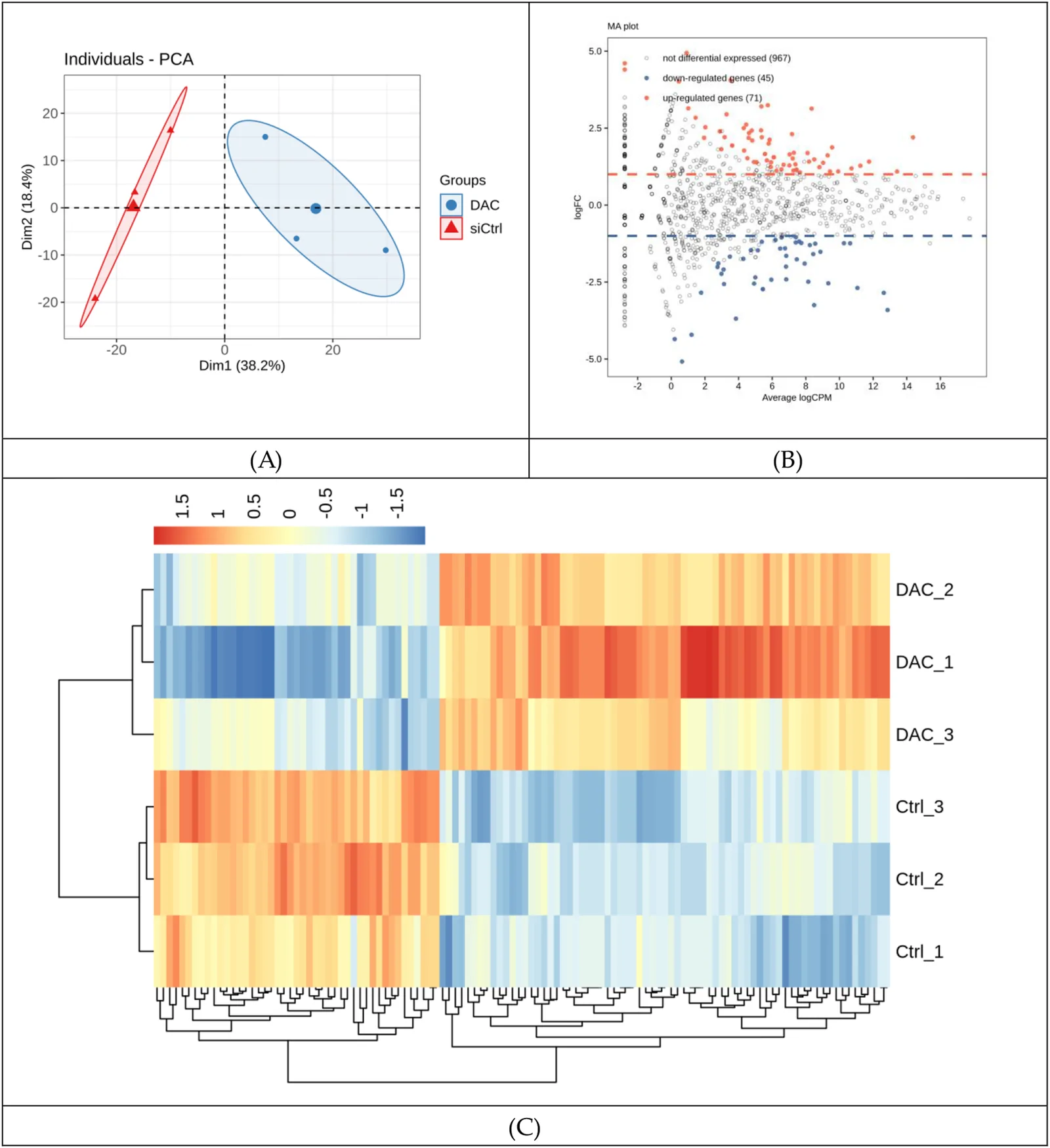
Transcriptome profiling of the miRNA expression changes in the GH3 cells after DAC treatment. **(A)** PCA based on the FPKM values of all detected miRNAs. The ellipse for each group is the confidence ellipse. **(B)** MA plot showing differentially expressed miRNAs between the DAC-treated and control groups. Upregulated miRNAs are labeled in red, whereas downregulated miRNAs are labeled in blue. **(C)** Hierarchical clustering heatmap showing the expression levels of all DEmiRNAs. Data were obtained from n = 3 independent biological replicates per group.

### 3.4 lncRNA–miRNA–mRNA network analysis in the GH3 cells after DAC treatment

After quantifying the mRNA, lncRNA, and miRNA expression levels in the cells before and after DAC intervention, we obtained DEmRNAs, DELncRNAs, and DEmiRNAs, respectively. Magia (integrated analysis tool) was used to conduct joint analysis of the interactions between the miRNAs and genes, and an interaction network map was drawn (**Figure 4A**). Lastly, the differentially expressed genes shared by the lncRNA–miRNA–mRNA network and correlated with one another were obtained: Fosl1, Nrbp2, Vash1, Ikzf3, Tpm2, Trim7, and their corresponding miRNAs and lncRNAs (**Figure 4B**).

**Figure 4:**
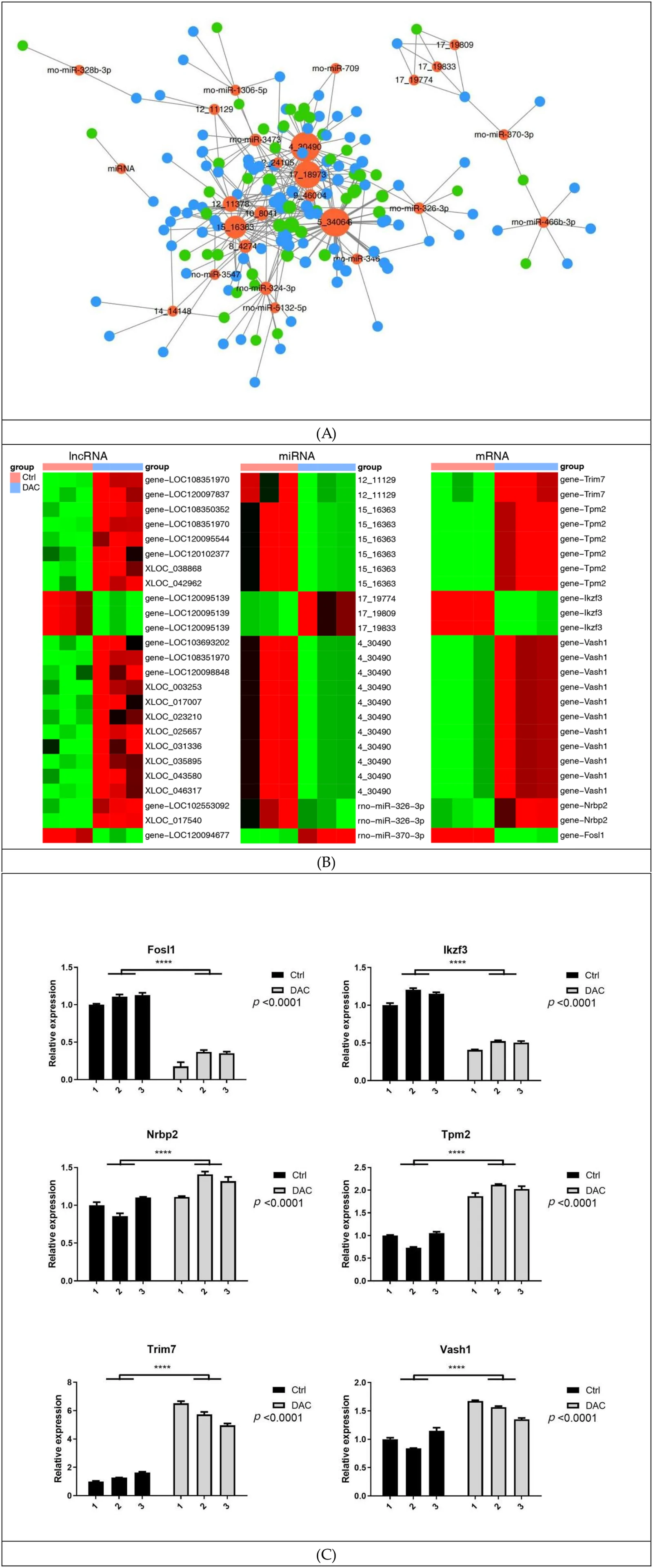
lncRNA–miRNA–mRNA network analysis in the GH3 cells after DAC treatment. **(A)** DElncRNA–DEmiRNA–DEG network for the GH3 cells after DAC treatment. **(B)** Heatmap showing the expression pattern and statistical difference of the DE-lncRNAs, DEmiRNAs, and DEmRNAs. **(C)** qPCR validation of the DEGs, including Fosl1, Ikzf3, Nrbp2, Tpm2, Trim7, and Vash1. Error bars represent the mean ± SEM. ****p < 0.0001.

The solution curve was analyzed by RT-qPCR, and the expression of the differential genes in the experimental group was detected by DEG analysis (**Figure 4C**).

### 3.5 Trim7 overexpression verification and cell proliferation and apoptosis experiments

Among the genes differentially expressed in GH3 cells following DAC treatment, Trim7 showed significant upregulation and was selected for further investigation. TRIM7 belongs to the tripartite motif (TRIM) protein family and has been implicated in the regulation of several biological processes, including cell proliferation, apoptosis, and immune responses (8). Previous studies have also reported roles for TRIM7 in tumor-related processes in several cancer types. For example, TRIM7 inhibits the progression of hepatocellular carcinoma by negatively regulating the malignant tumor protein Src (9), while Trim7 inhibits proliferation and triggers the apoptosis of non-small-cell lung cancer cells by downregulating miR-4491 (4). At the same time, studies have shown that the Trim7 gene subtype GNIP1 promotes GSK3β phosphorylation to regulate the degradation of β-catenin, thereby activating the Wnt/β-catenin signaling pathway in breast cancer cells (10). Thus, we hypothesized that Trim7 may be associated with the proliferation and apoptosis of GH3 rat pituitary tumor cells and their responsiveness to dopamine agonists.

Using the plasmid as a vector, Trim7 was overexpressed in GH3 cells, and the growth of the cells was recorded. RNA was extracted for qRT-PCR overexpression quality inspection, and the results indicated that the mRNA level was overexpressed 22–32-fold (**Figure 5A**). The Western blot method was used for gene overexpression quality inspection, and the results showed that there were differences between the NC and OE groups (**Figure 5B**).

**Figure 5:**
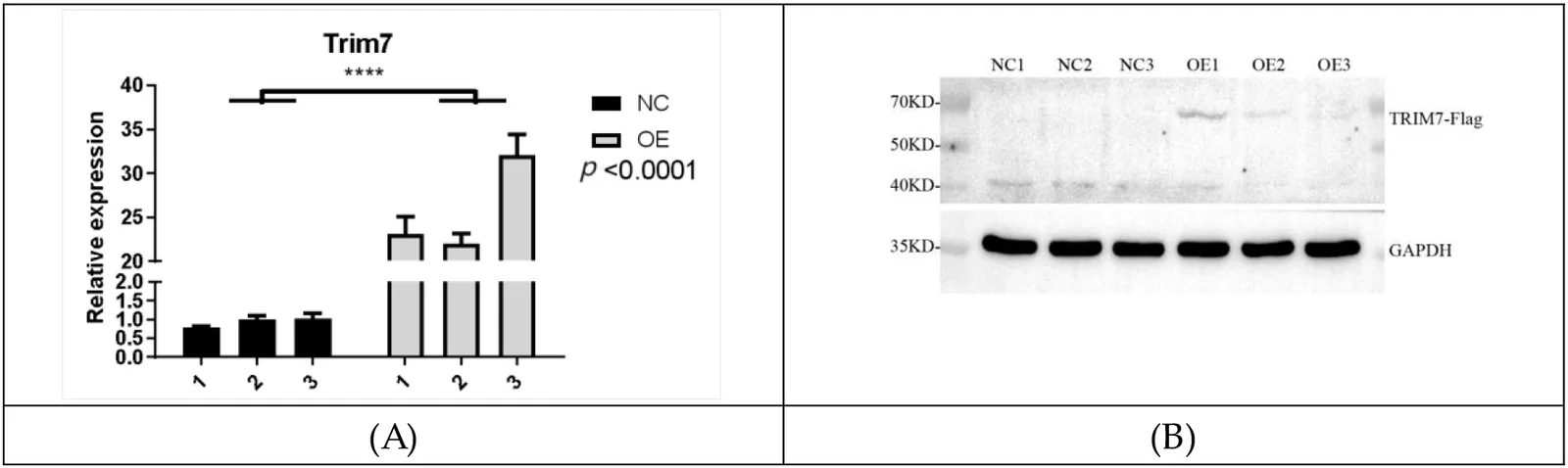
Trim7 overexpression and transfection quality verification. **(A)** qPCR validation of Trim7 after plasmid transfection. **(B)** Western blot validation of Trim7-FLAG protein expression after transfection. Data are presented as mean ± SEM, n = 3 independent biological replicates. Statistical significance was determined by two-tailed unpaired Student’s t-test. ****p < 0.0001.

The results of the cell apoptosis test showed that, in the GH3 cells, compared to OE–NC, 24 h of transfection with a further 24 h of 10 μM cabergoline treatment significantly promoted cell apoptosis, according to a t-test test (**Figure 6A**). The results of the cell proliferation test showed that, in the GH3 cells, compared to OE–NC, after 24 h of transfection with a further 24 h of 10 μM cabergoline treatment, OE–TRIM7 tended to inhibit cell proliferation, but the t-test showed no significance (**Figure 6B**).

**Figure 6:**
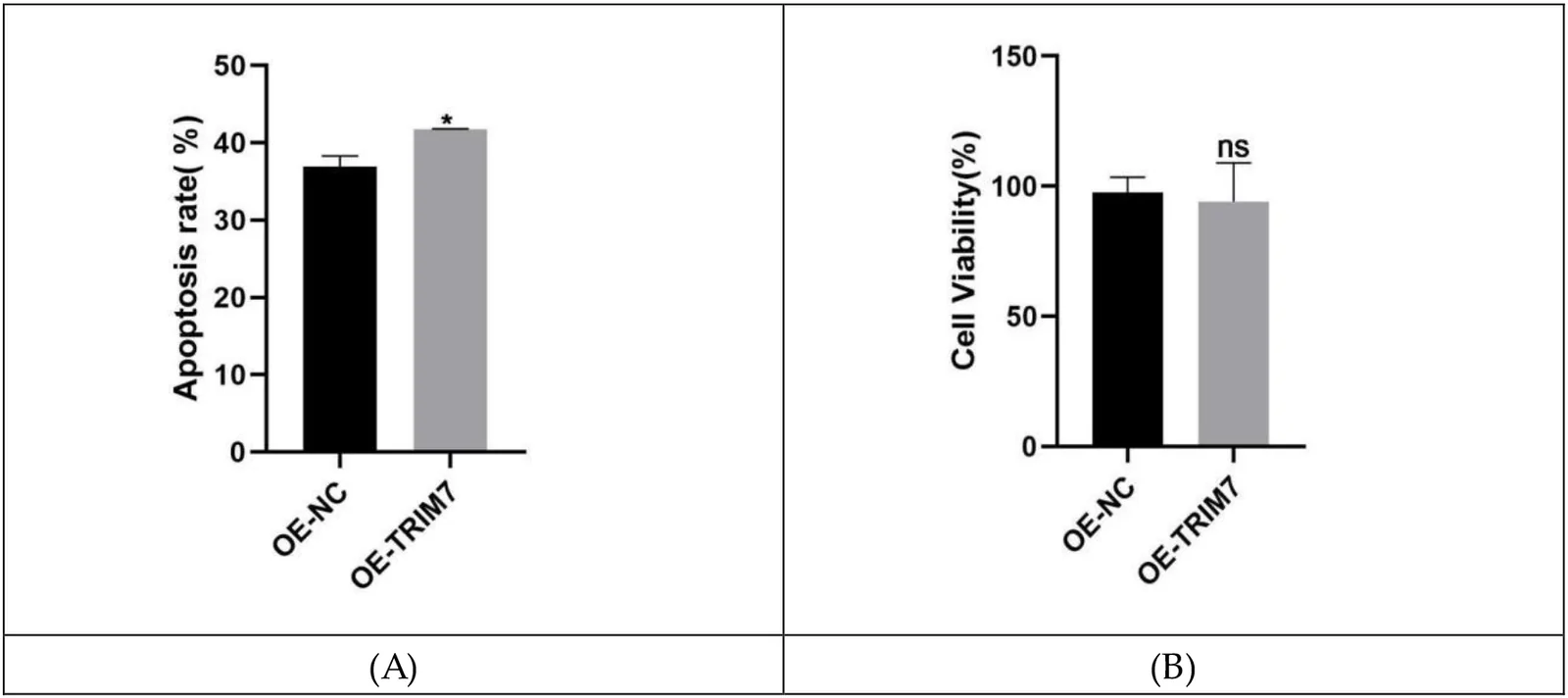
Cell proliferation and apoptosis assays. **(A)** Apoptotic rates of OE-NC and OE-Trim7 GH3 cells following 24 h transfection and subsequent 24 h treatment with 10 μM cabergoline. Elevated apoptosis was observed in the OE-Trim7 group. **(B)** Relative cell viability of OE-NC and OE-Trim7 GH3 cells after cabergoline treatment; a non-significant decreasing trend in cell viability was observed in OE-Trim7 cells. Data are presented as mean ± SEM, n = 3 independent biological replicates. Statistical significance was determined by two-tailed unpaired Student’s t-test. *p < 0.05, ns = not significant.

## 4 Discussion

For patients with prolactinomas, the preferred treatment is drug therapy. Generally speaking, the vast majority of patients with prolactinomas can achieve better therapeutic effects by taking dopamine receptor agonists, but there are still some patients in which the tumor cannot be well controlled, which is called drug-resistant prolactinoma. For such patients, the effect of non-drug treatment, including surgery and radiation therapy, is not satisfactory. This necessitates finding new treatment methods through drug means. Improving the sensitivity of DARPs to DAs is a very good research idea.

Histologically, DARPs show four types of changes: ① increased cellular atypia, ② increased cell proliferation activity, ③ increased tumor invasiveness, and ④ increased angiogenesis. The cause of these characteristic changes may be related to the inhibition of the dopamine D2 receptor (D2R) signaling pathway (11). Dopamine receptors are divided into types 1–5, and pituitary prolactinoma cells only express dopamine D2 receptors (D2Rs). When a D2R is not bound to a ligand, it couples with the Gα12 protein in the cell membrane, which can activate adenylyl cyclase, catalyze the conversion of adenosine into cAMP, and promote cell proliferation and the secretion of PRL. When D2Rs bind to dopamine from the hypothalamus or an exogenous dopamine agonist, they can inhibit Gα12 protein coupling in the membrane, inhibit the formation of cAMP, and eventually lead to ① a decrease in cell metabolism, ② downregulation of PRL gene transcription, and ③ cytoplasmic changes. The endoplasmic reticulum and Golgi apparatus shrink, the nucleus shrinks, the cell shrinks, and ④ cell death occurs (11).

There are several common mechanisms of DARPs. Firstly, the number of D2Rs in tumor cells decreases significantly. Studies have shown that the expression of D2Rs in the cell membrane of drug-resistant prolactinomas is only 60% of that observed in sensitive prolactinomas, and the transcriptional expression of D2R mRNAs in the cells is four times lower than that of the latter (12). Secondly, the expression of nerve growth factor receptor (NGFR) involved in the expression of D2Rs in tumor cells decreases. The activation of NGFR can upregulate the expression of D2Rs by activating NF-κB, and the expression of NGFR in DARPs decreases nearly 25-fold, resulting in a significant decrease in the expression of D2Rs (13). Thirdly, D2Rs exist in two alternately spliced isoforms, the long form (D2L) and the short form (D2S); the difference is the insertion of 29 amino acids into the third intracellular protein ring. These two D2Rs in tumor cells dysregulate the expression of subtypes. A study found that the ratio of D2S/D2L in DARPs is significantly lower than that in sensitive prolactinomas (14). Further studies have shown that a decrease in D2R expression may be related to the hypermethylation of its promoter CpG site, whereas it has nothing to do with the deletion or inactivation mutation of the gene (15). Accumulating evidence suggests that D2L and D2S may have different physiological functions. Iaccarino et al. (16) proved that D2S can inhibit the synthesis of prolactin and the proliferation of lactic acid bacteria cells, while D2L may promote the release of PRL. Furthermore, the expression levels of the D2R subtypes appear to affect the response of lactotroph adenomas to DA treatment. Caccavelli et al. (17) found that resistance to bromocriptine treatment appears to be related to the D2S/D2L ratio, with resistant patients having lower D2S/D2L ratios than responding patients. Passos et al. (18) reported high D2R expression in two patients with drug-resistant lactotroph adenomas, and they speculated that D2L may predominate over the D2S receptor subtype in these two patients. Some studies have also shown that the ratio of D2S/D2L is related to the responsiveness of prolactin to DA drugs, in which D2S plays an important role in the therapeutic effect of bromocriptine. Taken together, these data suggest that D2S plays an important role in inhibiting PRL synthesis and the growth of prolactinoma cells under DA treatment (14). Therefore, we have reason to believe that a certain drug may induce the demethylation of the D2R promoter CpG site, thereby upregulating the expression of D2Rs and reversing the sensitivity of DARPs to dopamine agonists.

In addition to the most important D2R receptor-related mechanism for the occurrence of DARPs, there are many potential mechanisms that can explain the resistance of pituitary lactotroph adenomas to DA drugs. The first mechanism is the distribution density of D2DR on the cell membrane. Studies have shown that, after *in vitro* culturing of tumor cells from patients with drug-resistant and sensitive lactotroph adenomas, related experiments confirmed that, compared to those from lactotroph adenomas sensitive to DAs, drug-resistant lactotroph adenoma cell membranes have only 10% of the density of dopaminergic binding sites found in the sensitive type, but their binding affinity is similar. The adenylyl cyclase activity in these cells, consistent with changes in the D2 binding site, was shown to be inhibited in 28.8% of DA-sensitive tumors and in 16.5% of DA-resistant tumors (19). The second mechanism is fibrosis in pituitary lactotroph adenomas. Bin Hu et al. (20) proposed that partially drug-resistant lactotroph adenomas show significantly more fibrosis than sensitive adenomas, which indicates that fibrosis may play a role in their drug resistance. In their study, 28 drug-resistant and 6 sensitive prolactinoma specimens were analyzed by Masson staining. The results showed that 42.8% (12/28) of the drug-resistant adenomas showed a high degree of fibrosis, and the average collagen content was 5.52% ± 0.95%. No obvious fibrosis was seen in any of the sensitive adenomas, for which the average collagen content was 1.45% ± 0.81%. The degree of fibrosis was also closely related to the reduction of D2DR.

According to the “central dogma”, gene expression is first transcribed from DNA to mRNA, which is translated into protein and exerts corresponding functions. Transcription factors bind to a gene’s promoter to initiate gene transcription. There is a CpG-rich region at the promoter site of a gene called a CpG island. Galdiner-Garden and Frommer first defined it as a CpG-rich region with a length of >200 bp and an actual/expected CG frequency of >0.6. The most notable feature of CpG islands is that they remain strictly unmethylated. These CpG islands are usually located at promoter alleles and are markers of gene activity (15). When the CpG site in the CpG island is over-methylated under the action of DNA methyltransferases (DNMTs) 1, 3a, and 3b, the expression of the gene can be silenced; that is, the expression level of the gene is reduced (21). However, the mechanism via which promoter methylation leads to gene silencing is unclear. The methylation of CpG sites is catalyzed by DNMT, and this process is also reversible. When the activity of DNMT is inhibited, the methylated site on the original promoter can be demethylated again, and the silenced gene can be expressed again (6).

Decitabine (5-aza-2 deoxycytidine, DAC), also known as 5-aza-2’-deoxycytidine, is a DNMT inhibitor that can inhibit promoter CpG island methylation. DAC is a natural 2′-deoxycytidine analogue of adenosine, which can be incorporated into DNA or RNA sequences during DNA replication and transcription in cells. It inhibits the action of DNA methyltransferase by forming a covalent compound with the latter, leading to demethylation of this sequence, thereby affecting cell differentiation and apoptosis (22). Consistent with this epigenetic property, our transcriptomic data showed that Trim7 expression was markedly upregulated after DAC treatment in GH3 cells. Nevertheless, we did not perform methylation analysis of the Trim7 promoter in the present study; therefore, we cannot conclude that the observed upregulation of Trim7 was specifically attributable to demethylation of its promoter region. Notably, the functional validation of Trim7 in this study was limited to overexpression-based gain-of-function experiments, without complementary loss-of-function experiments such as Trim7 knockdown or knockout. Therefore, the present findings do not establish Trim7 as a necessary mediator of the observed cabergoline-associated response, and additional loss-of-function studies are required to determine its causal contribution.

Previous research from our team showed that, in rat prolactinoma specimens, hypermethylation of the WIF-1 promoter −111 bp site was associated with decreased WIF-1 expression in tumor cells. In addition, treatment of MMQ cells with DAC increased WIF-1 expression. After treatment, the expression of WIF-1 increased by 6.56 ± 1.45 times (p < 0.001), and the cell proliferation was also significantly inhibited, reaching 37.8% ± 4.3% (p < 0.001) (7). In addition, we found that, after treating GH3 cells with different concentrations of DAC, the cell proliferation decreased in a concentration- and time-dependent manner. The GH3 cells were treated with 40 nM DAC for 72 h, and the inhibition rate of cell proliferation was the largest, reaching 87.6% ± 2.2%. After GH3 cells were treated with different concentrations of DAC for 72 h, SFRP4 protein expression increased in a dose-dependent manner, and the differences were statistically significant. Al-Azzawi found that the loss of D2R expression in GH3 cells is related to the hypermethylation of the promoter of its coding gene D2DR. After using DNMT inhibitors, the D2R expression in the GH3 cells was upregulated sixfold (8). Previous studies have suggested that DAC-associated upregulation of D2R may influence the activity of the canonical Wnt signaling pathway and may also be associated with cellular responsiveness to dopamine agonists. These observations raise the possibility that DAC-related molecular changes could contribute to the modulation of dopamine agonist responsiveness. However, the present study did not employ a validated dopamine agonist-resistant prolactinoma model; therefore, whether DAC can enhance or restore dopamine agonist sensitivity in resistant prolactinoma cells remains to be directly established. Further studies using validated dopamine agonist-resistant models are required to investigate this possibility and the underlying molecular mechanisms.

In this study, we constructed a lncRNA–miRNA–mRNA regulatory network using RNA-seq and miRNA-seq data, finally obtaining six target mRNAs (Fosl1, Trim7, Vash1, Tpm2, Ikzf3, and Nrbp2), and six groups of target genes were selected (miR-370-3p, Fosl1; miR-326-3p, Nrbp2; 4_30490, Vash1; 17_19809, Ikzf3; 15_16363, Tpm2; 12_11129, Trim7). Based on previous studies (4, 8–10), we hypothesized that Trim7 may be associated with the proliferation and apoptosis of GH3 rat pituitary tumor cells and their responsiveness to dopamine agonists. Therefore, in the present study, we overexpressed Trim7 in GH3 cells and subsequently treated the cells with cabergoline. Cell apoptosis and proliferation were evaluated by flow cytometry and CCK-8 assays, respectively. The results showed that cabergoline-treated OE-Trim7 cells exhibited significantly greater apoptosis than OE-NC cells. These data demonstrate a positive correlation between elevated Trim7 expression and enhanced cabergoline-associated apoptosis in GH3 rat pituitary tumor cells. Collectively, Trim7 represents a candidate factor warranting further investigation in the context of dopamine agonist responsiveness.

Although we obtained cross-comparison results after gene sequencing, as well as used bioinformatics tools for comparison and verification, from the perspective of scientific practice, further experimental verification is still needed, including but not limited to human tissue cytology verification, animal model verification, and animal cytology verification. At present, there are many related oncological studies on the Trim7 gene, including the proliferation, invasion, and migration of osteosarcoma cells and the migration and invasion of breast, lung, and pancreatic cancer cells. Correlations in biological processes such as invasion, migration, drug resistance, and apoptosis support the research value of drug-resistant prolactinoma.

At the same time, studies have found that the methylation of the Wnt pathway inhibitors WIF-1 and SFRP4 leads to a downregulation of the expression of the corresponding genes, thereby activating the Wnt canonical pathway and promoting the formation of prolactinomas (23). We speculate that the reason for the characteristic changes in prolactinomas may be related to the activation of some proliferation-related pathways such as the Wnt pathway. The Wnt pathway participates in at least three axes. The first axis is the classic Wnt/β-catenin pathway, in which Wnt ligands interact with their receptors to inhibit the hydrolysis of β-catenin, such that β-catenin can activate target genes, regulating cell proliferation and inhibiting apoptosis. β-catenin is the main effector protein of this pathway, and the transfer of β-catenin from the cytoplasm to the nucleus is the main feature of the activation of the canonical Wnt/β-catenin pathway. The canonical Wnt/β-catenin pathway plays an important role in embryonic development and organ formation. It is an important regulator of stem cells and has an important relationship with the occurrence of various tumors, including pituitary tumors. The second axis is the non-canonical Wnt/JNK pathway, also known as the Wnt/PCP pathway. The third axis is the non-canonical Wnt/Ca2+ pathway, which binds Wnt to Fzd receptors, leading to a Ca2+ influx, which activates CamKII and PKC in a G-protein-dependent manner (24). Non-canonical pathway Wnt ligands do not require β-catenin to function. Wnt pathway inhibitors include two types. The first type can block Wnt binding to its receptors by binding to them. The second type blocks Wnt binding to the its receptors by directly binding to Wnt ligands. This type includes WIF-1 and SFRP (Secreted frizzled-related protein family). Upon binding to Wnt, binding to the receptor is inhibited, thereby blocking the canonical Wnt/β-catenin pathway. A previous study used gene chip technology to detect and verify prolactinoma specimens and normal human pituitary specimens (25), revealing that the genes related to the Wnt pathway were significantly changed in prolactinomas. Therefore, we can infer that the Wnt pathway may be related to lactation, involved in the occurrence and development of adenomas. In a rat prolactinoma model, it was found that the expression of WIF-1 in the tumor group was significantly decreased, and its mRNA expression was found to be negatively correlated with the methylation level of the −111 bp CpG site (y = 5.302 − 17.342x (R = 0.949, p = 0.001), where y is the relative expression level of WIF-1 mRNAs and x is the degree of methylation of the WIF-1 promoter region) (23). Studies have also found that the expression of the SFRP 4 protein in invasive pituitary adenomas was significantly lower than that in noninvasive pituitary adenomas (p < 0.001), while the methylation level of the CpG site −62 bp upstream of its promoter,in the invasive group reached 29.4% ± 2.6% expression (n = 20), which was significantly lower than that of the noninvasive group (15.5% ± 1.6% vs. 7.63% ± 1.8%, p = 0.031, n = 20 vs. 8) (26). In conclusion, the methylation of the promoters of the Wnt pathway inhibitors WIF-1 and SFRP4 leads to the downregulation of the expression of the corresponding genes, thereby activating the canonical Wnt pathway and promoting the formation of prolactinomas. At the same time, the D2R receptor is a G-protein-coupled signal transduction pathway. For a long time, it was considered to be a completely different pathway from the Wnt classic pathway, with seemingly no connection between the two. However, it has been found that the use of antipsychotic drugs against D2Rs can upregulate the expression levels of molecules downstream of the Wnt classic pathway, such as Dvl, GSK-3β, and β-catenin. Further studies have found that D2Rs can directly interact with β-catenin below the level of GSK 3-β, resulting in a decrease in the distribution of the latter in the nucleus, thereby inhibiting LEF-1-dependent transcriptional activity in the Wnt pathway. This shows that the D2R signaling pathway and the Wnt canonical pathway communicate with one another through β-catenin (crosstalk) (27). Therefore, we further envisage that DAC can be used to treat DARPs, inducing the latter to express WIF-1 and SFRP4, which can achieve antitumor effects by inhibiting the Wnt canonical pathway; however, this also needs further study. Several limitations of the current study need to be addressed. All epigenetic and functional assays in this work were performed solely in GH3 rat pituitary tumor cells, without human tumor specimens or clinical cohort analyses. For this reason, we cannot provide definitive clinical administration recommendations for all drug-resistant prolactinoma patients based solely on the present *in vitro* findings. Prior to translating these observations into clinical practice, further validation using human prolactinoma tissues and prospective clinical studies will be required to determine whether Trim7 expression is associated with treatment responsiveness and to investigate whether Trim7 methylation status has potential biomarker value.

## 5 Conclusions

Our cellular experiments demonstrated that decitabine upregulates Trim7 expression in GH3 rat pituitary tumor cells, and elevated Trim7 levels correlate with enhanced cabergoline-mediated apoptosis. These gain-of-function findings identify Trim7 as a candidate factor associated with cellular responsiveness to cabergoline. Further studies using loss-of-function approaches and validated dopamine agonist-resistant models are required to determine the causal role of Trim7 and the potential relevance of these findings to drug-resistant prolactinomas.

## Data Availability

The datasets presented in this study can be found in online repositories. The names of the repository/repositories and accession number(s) can be found below: https://www.ncbi.nlm.nih.gov/, &z.cirf; PRJNA1120190 https://www.ncbi.nlm.nih.gov/, &z.cirf; PRJNA1119261. PRJNA1120190 : Trim7 expression regulates the Wnt/b-catenin signaling pathway involved in the mechanism of drug resistance in prolactin adenoma DAs PRJNA1119261 : Mechanistic study of DAC synergizing with cabergoline in the treatment of drug-resistant prolactinomas.
