# Supplementary material for: Decitabine modulates dopamine agonist sensitivity in gh3 rat pituitary tumor cells via Trim7
Source: Front Endocrinol (Lausanne). 2026 Sep 17;17:1857666. doi: 10.3389/fendo.2026.1857666 (PMC13629320; doi:10.3389/fendo.2026.1857666)
Supplement: Supplementary file 1 [file Table1.docx]

**Table 1 Primers Information**

| Gene | Primer | Sequence (5'-3') | PCR Products |
| --- | --- | --- | --- |
| Rat GAPDH | Forward | TGCCACTCAGAAGACTGTGG | 129bp |
|  | Reverse | TTCAGCTCTGGGATGACCTT |  |
| Trim7 | Forward | GGAGCAGTGTCCTTCTATG | 117bp |
|  | Reverse | TCTCAAGTAGGTGCCAGTA |  |
